# Italian Expert Consensus on Women’s Nutrition Across the Life Course: A Modified Delphi Study

**DOI:** 10.3390/nu18071053

**Published:** 2026-03-26

**Authors:** Laura Sarno, Dario Colacurci, Maurizio Guida, Rossella Elena Nappi, A.G.U.I.

**Affiliations:** 1 Department of Neurosciences, Reproductive Science and Dentistry, University Federico II, 80131 Naples, Italy; laura.sarno@unina.it (L.S.); maurizio.guida@unina.it (M.G.); 2 Department of Public Health, School of Medicine, University of Naples Federico II, 80131 Naples, Italy; 3 Department of Clinical, Surgical, Diagnostic and Pediatric Sciences, University of Pavia, 27100 Pavia, Italy; r.nappi@smatteo.pv.it

**Keywords:** women’s nutrition, micronutrient supplementation, personalized nutrition, delphi consensus, life-course approach

## Abstract

**Objective:** Nutrition is a key determinant of women’s health across all life stages. Clinical practice remains heterogeneous because of lack of evidence and non-homogeneous guidelines. Despite growing research on micronutrient supplementation, skeptical opinions persist around universal versus individualized approaches, optimal dosages, and life-stage-specific recommendations. **Material and methods:** This is a modified Delphi process conducted under the supervision of the Italian Association of University Gynecologists and Obstetricians (AGUI). Thirteen Italian experts in gynecology and obstetrics completed two rounds of anonymous online surveys (September–November 2025). The questionnaire, developed through a scoping review, covered six domains: pre-/periconception, pregnancy, postpartum, routine supplementation in non-pregnant women, nutrition in gynecological conditions, and menopause. Consensus was defined as ≥75% agreement on a 10-point Likert scale. Quantitative data were summarized descriptively, and qualitative comments contextualized findings. **Results:** Experts strongly supported personalized nutritional strategies across all life stages. Consensus was reached on individualized micronutrient supplementation in the preconception period and on the prescription of active folates for women undergoing assisted reproduction. In pregnancy, agreement emerged for universal DHA supplementation (200–300 mg/day); however, universal vitamin D supplementation lacked consensus except in gestational diabetes. In the postpartum period, iron supplementation for non-breastfeeding women reached consensus, while micronutrient recommendations for breastfeeding women remained uncertain. Strong agreement supported personalized dietary approaches for PCOS, endometriosis, and gestational diabetes, including inositol use, while evidence for interventions in severe premenstrual syndrome remained insufficiently supported. In menopause, consensus was reached for macronutrient adjustments and universal calcium and vitamin D supplementation. **Conclusions:** This Delphi consensus highlights shared expert perspectives on nutritional care in women and identifies key evidence gaps, particularly regarding vitamin D in physiological pregnancy, postpartum micronutrient needs during breastfeeding, and nutritional strategies for premenstrual disorders. Unified life-course guidelines and future research on standardized nutritional assessments are necessary for nutritional approach management.

## 1. Introduction

Prevention of principal diseases and early evaluation of pathological conditions are the standards of care in modern medicine [1]. Nowadays, clinicians aim to create the best conditions for patients, to prevent adverse outcomes. For this reason, nutrition is becoming a crucial determinant of human health throughout the life course, from adolescence to old age [2]. The impact of different dietary patterns and micronutrient supplementation has been examined across gynecologic conditions [3] from prepuberal to post-menopausal age [4,5,6,7]. Every phase of a woman’s life is characterized by specific stages with unique nutritional demands and vulnerabilities. Despite increasing evidence on the role of micronutrients, significant uncertainties remain. Clinicians disagree and give conflicting opinions about which nutrients should be prioritized, when they should be supplemented, at what doses, and in which subgroups individualized interventions are needed [8]. Although well-established micronutrients, such as folic acid [9], iron [10], iodine [11], vitamin D [12], docosahexaenoic acid [13], and calcium [14], are widely discussed in obstetric and gynecological contexts, recommendations vary substantially among clinicians. The role of supplementation in both physiological and pathological conditions remains widely debated. Indeed, transparent, harmonized, and transversal guidelines for nutritional management throughout the female life course are still lacking, although evidence is expanding [15,16,17,18,19,20,21]. In Italy, as in other European contexts, current guidelines and expert consensus are primarily pregnancy-focused [22] and fail to comprehensively address the nutritional needs of women along the life course. This leads to significant variability in clinical practice among specialists and centers, with potential risks of under- or over-supplementation, inconsistent adherence, and increased costs. Furthermore, the risk–benefit balance between universal supplementation and individualized approaches remains uncertain, particularly when nutritional biomarkers are not routinely assessed. Most available studies and clinical guidelines primarily focus on pregnancy-related nutrition, while comprehensive and integrated recommendations addressing adolescence, reproductive age outside pregnancy, gynecological conditions, postpartum, and menopause are still limited. As a consequence, clinical practice is often based on heterogeneous applications of the available evidence; indeed, even experts in the field may adopt different approaches to nutritional supplementation. So, the Delphi method could be a valid tool to underscore areas of uncertainty. Therefore, the aim of the present study was not to replace existing evidence syntheses, but rather to complement the current literature by assessing the level of agreement among experienced clinicians on key aspects of nutritional management across the female life course and by identifying priority areas for future research and guideline development. Given this context, a well-structured, evidence-based expert consensus is necessary to outline shared recommendations on several unsolved questions: when a balanced diet alone may suffice; when specific nutritional interventions or general supplementation are appropriate; when and how personalized supplementation should be carried out (including serum monitoring and timing); the suitability of food fortification; the clinical importance and ideal duration of postpartum supplementation; and key micronutrients for each life stage. These points were integrated into the current Delphi-method consensus.

The Delphi method is advantageous in contexts where scientific evidence is divergent or incomplete, as it enables the convergence of multidisciplinary expertise to inform clinical practice. Unlike previous Delphi studies that mainly addressed the maternal–fetal period, this project broadens the scope to include women’s nutrition throughout their entire life cycle, covering less-studied areas. The main goal is to develop shared, practical recommendations for gynecological practice while pinpointing knowledge gaps and research priorities. In a previous article, conducted under the supervision of the Italian Association of University Gynecologists and Obstetricians (AGUI), we evaluated a cohort of trainees from the same geographical area to assess their level of knowledge regarding nutrition across the different stages of a woman’s life [23]. In this occasion, always under the supervision of AGUI, this study used a modified Delphi process with Italian experts in gynecology and obstetrics with proven clinical and scientific expertise to assess the level of consensus on dietary and supplement use across the main stages of a woman’s life; determine when to favor universal versus individualized approaches and which clinical or biochemical markers may guide them; and propose harmonized, evidence-informed, and feasible recommendations for clinical practice, laying the groundwork for future evidence-based updates.

## 2. Materials and Methods

### 2.1. Study Design

This study used a modified Delphi approach that adheres to specific standards for expert consensus-building for healthcare research. The Delphi process is particularly suitable for topics where high-quality evidence may be limited or fragmented, and where expert opinions are vital for guiding informed clinical decisions. Delphi studies in the health sciences are widely used to obtain consensus among experts on complex clinical topics and typically involve iterative survey rounds with structured feedback to facilitate convergence of expert opinion [24]. In this context, the current Delphi study aimed to explore, understand, and establish expert opinions on nutritional management of women at different stages of life, including adolescence, reproductive years, pregnancy, postpartum, and menopause, with a specific focus on micronutrient supplementation, dietary patterns, and individualized approaches in both physiological and pathological conditions. The study was carried out in two survey rounds, conducted between 29 September 2025 and 21 November 2025, via an anonymous Google Form. Conducting at least two rounds with structured feedback is considered a core methodological feature of Delphi studies and allows participants to reconsider their responses in light of aggregated group opinions [24]. Each round was designed to collect agreement on points of divergence between experts progressively. Responses were anonymized to prevent bias from hierarchical status or expertise. The current study was carried out under the supervision of the Italian Association of University Gynecologists and Obstetricians (AGUI) and guided by a steering committee composed of expert clinicians and researchers with recognized expertise in gynecology, obstetrics, and nutrition. AGUI is the national scientific society representing academic gynecologists and obstetricians in Italy, and its involvement ensured methodological oversight and the selection of panelists with recognized academic expertise in women’s health. The study design followed the principles of the Declaration of Helsinki. All participants provided voluntary consent to participate. Responses were collected anonymously, and no personal identifiers were stored at any stage of data collection. No sensitive personal data was requested or stored, and all procedures ensured full compliance with applicable privacy regulations.

### 2.2. Questionnaire Development

The Delphi questionnaire was developed through a structured, multistep process informed by the following:

1. A scoping review of international and national guidelines on women’s nutrition and micronutrient supplements (including WHO [25], EFSA [26], and the Italian Ministry of Health recommendations [27];
2. Analysis of past Delphi consensus statements on maternal nutrition (e.g., Cetin et al. [28]);
3. Expert discussions within the committee to adapt the content.

The final questionnaire was structured with six thematic sections, which covered the main stages and conditions of a woman’s life:

1. Pre-/Periconception: Awareness and management of nutritional needs, role of specific supplementation (folates, vitamin D, iron, iodine, DHA), and individualized as opposed to universal strategies, including assisted reproductive technologies (ART).
2. Pregnancy: Adequacy of nutrients across trimesters, timing and dose of vitamin D and DHA, consideration of the necessity of serum monitoring, and management of pregnancies complicated by obesity, diabetes, or previous hypertensive disorders.
3. Postpartum (lactating and non-lactating women): Priority micronutrients (folates, DHA, selenium, calcium, magnesium, iodine, vitamin K, vitamin D, B-group vitamins, iron) and duration of supplementation (1 month, 3 months, 1 year).
4. Routine supplementation in non-pregnant women: Iron management in adolescents with heavy menstrual bleeding, early nutritional intervention for bone health, and evaluation of a cyclical dietary program based on hormonal phases.
5. Nutrition in gynecological conditions: Individualized nutritional therapy and supplement strategies in endometriosis, polycystic ovary syndrome (PCOS), severe premenstrual syndrome, and discussion on the role of inositols in PCOS, idiopathic infertility, and gestational diabetes.
6. Menopause and hormonal contraception: The role of phytonutrients (e.g., isoflavones, flavonoids), macronutrient adjustments in diet, cognitive protection, and possible supplementation for women on combined hormonal contraceptives or folate-fortified formulations.

Every question was framed as a declarative statement, and examiners expressed their level of agreement using a 10-point Likert scale (0–9), where 0–5 indicated increasing disagreement, 6 indicated a neutral position, and 7–9 indicated increasingly strong agreement. Moreover, for selected domains, additional responses were included (e.g., dosage options and supplementation duration). Every question also provided an open-ended response to allow experts to justify their opinions. The full set of questions administered during the two Delphi rounds is available in the Supplementary Materials (Supplementary Materials S1: Round 1 questionnaire; Supplementary Materials S2: Round 2 questionnaire).

### 2.3. Panel Recruitment

A total of 13 Italian experts in gynecology and obstetrics were invited to participate, selected according to their documented clinical and scientific expertise in women’s health and nutrition. Panel recruitment was coordinated by an AGUI-appointed scientific committee, which defined the panel composition and eligibility criteria before the start of the Delphi process. The inclusion of experts was exclusively from Italian academic settings, as the objective of this study was to assess the perspectives of university-based specialists within the Italian healthcare and academic context.

The committee established that a panel of 13 experts would be appropriate for the Delphi process, balancing methodological feasibility, high-level expertise, and geographic representation across Italy. Participants were invited by email with a statement of information that outlined the aims of the study, the estimated time required, and assurances of anonymity. Participants were not rewarded with financial incentives. Participation was voluntary, and implied consent was obtained before conducting the survey.

Panelists were selected according to the following criteria: (i) senior academic expertsin gynecology and obstetrics at an Italian university; (ii) documented clinical and scientific expertise in women’s health, nutrition, or related research fields; and (iii) representation of different geographic areas of Italy.

Panelists were geographically distributed across Italian academic and hospital centers, ensuring representation from both clinical and research settings. Thirteen experts were ultimately invited in order to ensure balanced representation across Northern, Central, and Southern Italy. All invited experts accepted participation in the study; therefore, no further recruitment was required. The panelist institutional affiliation are showed in Table 1. Their responses were collected independently and securely through an online survey platform.

**Figure 1:**
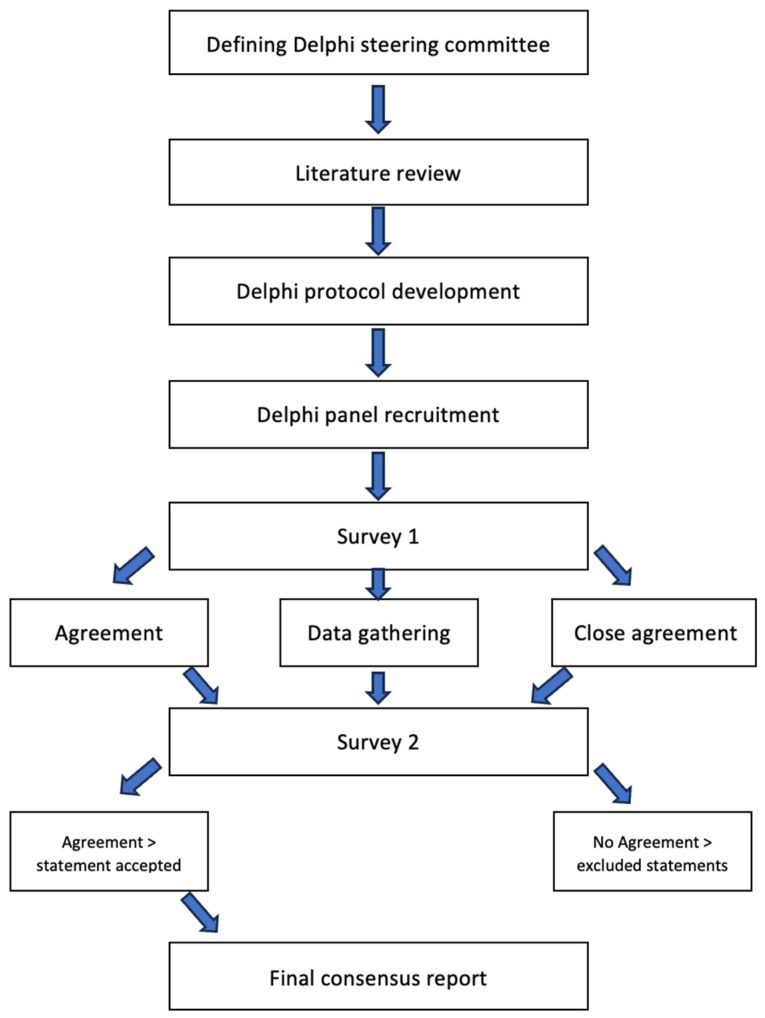
Overview of the modified Delphi process.

Figure 1 shows the flowchart of the panel recruitment.

### 2.4. Delphi Process

This was the Delphi consensus with two rounds:

- Round 1: Experts were provided with the preliminary form of the questionnaire, and they gave quantitative responses and qualitative opinions for every question. Responses were analyzed to determine levels of agreement and identify items requiring further clarification.
- Round 2: Aggregated and anonymized outcome scores from Round 1 were provided to the experts for each question. Median scores, interquartile ranges (IQRs), and summary comments were included. They were then asked to reconsider or confirm their responses in light of the group feedback.

This iterative approach enabled reflection and convergence toward a consensus while respecting individual autonomy.

### 2.5. Consensus Definition and Analysis

Consensus thresholds were predefined before data collection, as reported below:

- Consensus agreement was achieved when ≥75% of the respondents rated an item between 7 and 9 on the Likert scale.
- Consensus disagreement was defined as ≥75% rating between 0 and 5.
- Near-consensus was defined as 69–74% agreement or disagreement.
- Items not reaching consensus after two rounds were considered areas of uncertainty or priority for future research.

Consensus was defined as ≥75% agreement among panelists. Given that the Delphi panel consisted of 13 experts, this threshold corresponded to agreement from at least 10 panelists for a statement to be considered as having reached consensus. The ≥75% agreement threshold was chosen because methodological syntheses [24] show that health-science Delphi studies most commonly define consensus using percentage agreement and frequently adopt cut-offs between 70% and 80%; therefore, 75% represents a widely used balance between methodological rigor and feasibility.

Descriptive statistics (median, IQR, percentage of agreement) were employed to analyze the responses. Qualitative opinions were analyzed to contextualize quantitative findings or to clarify gaps. Given the nature of this study, which aimed to gather expert opinions, inferential statistical analysis was not warranted.

### 2.6. Ethical Considerations

The study was conducted in accordance with the principles of the Declaration of Helsinki and current Italian privacy regulations, including Regulation (EU) 2016/679 (General Data Protection Regulation, GDPR UE 2016/679) [29]. All data were collected anonymously, and participants could withdraw at any stage without justifying. This study protocol was peer-reviewed by the AGUI scientific board and approved for exemption from a full ethics review, as it was a survey for expert review and did not involve patient treatment or the collection of sensitive data.

## 3. Results

### 3.1. Panel Characteristics

Thirteen Italian experts in gynecology and obstetrics participated in the Delphi panel. All had documented clinical and/or academic experience in women’s nutrition and reproductive health.

All experts were affiliated with 13 university centers across Italy. The geographic distribution ensured full national representation, with five centers located in Northern Italy, 3 in Central Italy, and 5 in Southern Italy. All panelists had documented clinical and/or academic experience in women’s nutrition and reproductive health. The response rate was 100% for both Round 1 and Round 2.

All panelists had over 10 years of clinical experience, and several had contributed to national guidelines or position statements on women’s health.

### 3.2. General Perspectives on Women’s Nutrition

Most panelists (61.5%) agreed that there is insufficient awareness and education among specialists regarding nutrition. They pointed out the lack of attention from both healthcare professionals and patients. Given the rising rates of obesity and the low nutritional awareness during the preconception phase, many experts stressed the importance of including structured nutritional counseling in routine gynecologic care, starting in adolescence and continuing through menopause.

### 3.3. Pre-/Periconceptional Period

The preconception phase is a crucial window for nutritional intervention. Experts strongly agreed on personalized supplementation based on each patient’s dietary deficiencies (84.6%). The majority did not recommend generic supplementation. Panelists commented that tailored nutritional support based on the patient’s characteristics is the preferred approach, but it is not always feasible.

Consensus on folate supplementation in patients undergoing Assisted Reproductive Techniques (92.3%) was achieved. The experts advised prescribing folates in their active form, especially in the presence of documented hyperhomocysteinemia. However, no consensus was reached regarding the dosage of folate supplementation, although 61.5% of panelists supported routine supplementation with 400 µg/day of folate, consistent with the dosage commonly recommended for spontaneous pregnancy.

### 3.4. Pregnancy

Regarding physiological pregnancies, there was no consensus that diet alone is sufficient to meet all nutritional requirements during the second and third trimesters. However, most experts (53.8%) agreed that diet alone is insufficient. The same result was found for general supplementation during these pregnancy phases. Consensus was reached (76.9%) on personalized supplementation based on each patient’s dietary deficiencies. 92.3% of panelists agreed that, even in physiological pregnancies, personalized nutritional patterns should be considered based on individual factors such as pre-pregnancy BMI, inflammatory profile, microbiota, and so on. In the second round of the consensus, panelists finally agreed on DHA supplementation during pregnancy for all women, starting from the first trimester, with a standard dosage of 200–300 mg/day, adjusted only in cases of high fish consumption or specific risk factors (consensus 76.9%), with side effects of little clinical significance (consensus 76.9%). Conversely, the second round of the consensus showed no agreement on universal vitamin D supplementation during pregnancy from the first trimester, although most experts (61.8%) supported a 2000 IU/day dosage except under special conditions, such as extreme BMI, seasonality, and lifestyle. Regarding pathological pregnancy conditions, consensus was reached to recommend vitamin D supplementation (1000–2000 IU/day) in women with gestational diabetes mellitus, regardless of initial serum levels, to support metabolic control and maternal–fetal well-being (consensus 76.9%), but not in women with a history of gestational hypertension (36.5%). The lack of consensus regarding universal vitamin D supplementation during pregnancy likely reflects the ongoing debate in the literature and the variability in clinical practice. While vitamin D is widely recognized as essential for fetal skeletal development, calcium metabolism, immune regulation, and placental function, the evidence supporting routine universal supplementation remains heterogeneous. During the Delphi process, several experts underscored that the available literature does not consistently support a universal supplementation approach and emphasized that vitamin D supplementation may be more appropriately guided by individual risk factors. Panelists suggested that factors such as maternal BMI, lifestyle, seasonality, and baseline vitamin D status should be considered when deciding if supplementation is required.

### 3.5. Postpartum

The expert committee principally focused on micronutrient supplementation during this phase of women’s lives, differentiating between women who are breastfeeding and women who are not breastfeeding. Considering multivitamin supplementation in the postpartum period for women who are not breastfeeding, a consensus was reached for iron supplementation (84.6%) for at least 3 months after delivery, adjusting the dosage based on blood values or symptoms of deficiency. For the same population, experts found agreement about the uselessness of selenium, DHA, and vitamin K supplementation. Moreover, considering the population of women who are breastfeeding, a proper consensus was not found for any micronutrients. A close consensus (69.2%) was reached for folate, B-group vitamins, and iron supplementation. Vitamin D may also play an important role during the postpartum period. Adequate vitamin D status contributes to calcium absorption and maternal bone recovery after pregnancy and lactation, while also supporting immune function. Emerging evidence suggests that vitamin D may influence mood regulation, with some studies indicating a possible association between vitamin D deficiency and postpartum depressive symptoms. In addition, maternal vitamin D status can affect vitamin D content in breast milk and may therefore influence infant vitamin D status and early skeletal development. Despite these potential benefits, the available evidence remains heterogeneous regarding optimal supplementation strategies during the postpartum period, which may partly explain the variability in expert opinions observed in the Delphi panel.

### 3.6. Routine Supplementation in Non-Pregnant Women

A total of 84.6% of panelists agreed about iron supplementation (or targeted dietary increases) for adolescents with heavy menstrual bleeding, even in the absence of anemia, possibly based on periodic blood count screening. On the other hand, only a near consensus (69.2%) was reached to recommend early nutritional interventions to promote bone health before age 30. This recommendation primarily refers to nutritional strategies aimed at optimizing peak bone mass during early adulthood, including adequate intake of calcium, vitamin D, and sufficient dietary protein, as well as lifestyle factors such as regular physical activity, which are recognized as key determinants of long-term bone health. The “cyclical” nutritional approach, i.e., modulation according to hormonal phases of the menstrual cycle (e.g., follicular vs. luteal phase), was found not acceptable.

### 3.7. Nutrition in Gynecological Conditions

The first round of the Delphi Consensus showed immediate agreement around supplementation in specific gynecological conditions. There was a defined consensus about nutritional personalization in the management of Polycystic Ovary Syndrome (PCOS). Experts’ comments emphasized the importance of weight control and the necessity of reducing or eliminating sucrose, reducing high-glycemic index carbohydrates, increasing fiber and healthy protein intake, and consuming a protein-based breakfast. Moreover, a consensus of 92.3% was found for the supplementation of inositol in PCOS dietary routine, as well as in Gestational Diabetes patients. The second round of the survey showed consensus (84.6%) for the management of endometriosis patients: it was considered useful to recommend a personalized nutritional approach in women with endometriosis, aimed at reducing inflammation and improving gastrointestinal symptoms and pain, potentially involving a specialized nutritionist. Conversely, no consensus (69.2%—close consensus) was found to recommend personalized nutrition for women with severe premenstrual syndrome, aimed at modulating symptoms through inflammation control and improving metabolic and hormonal balance.

### 3.8. Nutrition in Menopause

According to the preexisting literature [30], experts found agreement for this category of patients about a modification of caloric intake and macronutrients to account for changes in body fat distribution. Calcium and Vitamin D are the only micronutrients that reached consensus among the panelists as universal supplementation in post-menopause. No consensus was found on omega-3 and antioxidant supplementation as a preventive measure for cognitive and neurological health in menopausal women, nor on a magnesium supplementation diet.

### 3.9. Consensus

**Figure 2:**
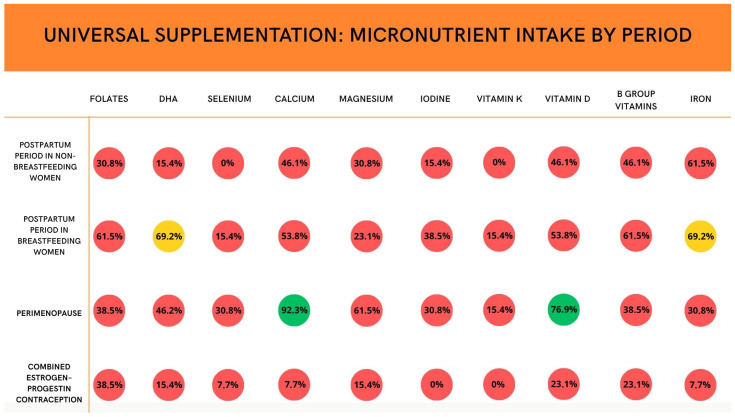
Levels of consensus on universal micronutrient supplementation in postpartum, perimenopause and hormonal contraception. Color coding: green represents consensus (≥75% agreement among panelists), yellow represents near consensus, and red represents no consensus.

**Figure 3:**
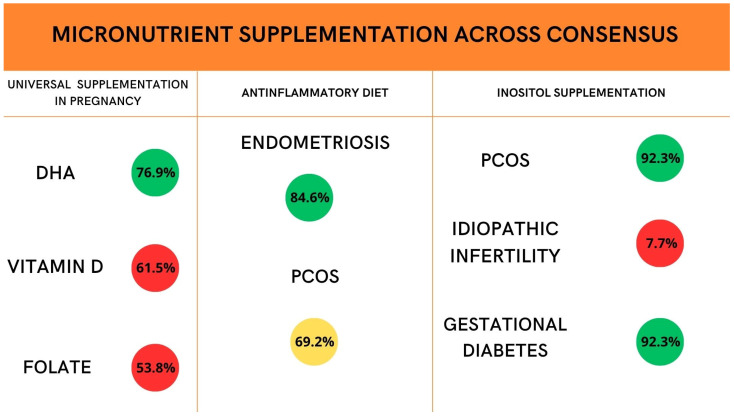
Levels of expert consensus on micronutrient supplementation across different clinical contexts. Color coding: green represents consensus (≥75% agreement among panelists), yellow represents near consensus, and red represents no consensus.

Figure 2 summarizes panelists’ agreement about universal supplementation of different micronutrients in different phases of women’s lives. Figure 3 shows levels of expert consensus on micronutrient supplementation across different clinical contexts. Table 2 summarizes all recommendations emerging from the Delphi consensus on women’s nutrition across the reproductive lifespan.

## 4. Discussion

The Delphi method provides a forum for discussion and collaboration among experts in a specific area to identify gray areas of recommendations, prompting scientific committees to develop guidelines and research to investigate these gaps. The panelists in this Delphi consensus recognize the importance of nutrition throughout the female life course and the persistent fragmentation of clinical practice in this area. Although there is clear evidence supporting the role of diet and micronutrient supplementation in reproductive medicine, pregnancy, and routine care, the lack of unified guidelines continues to obstruct consistent, high-quality nutritional counseling in gynecology and obstetrics. This study, involving experienced Italian clinicians and researchers, provides a structured synthesis of expert perspectives and reveals both areas of agreement and domains requiring further scientific investigation. A central finding emerging from this panel is the strong need for personalized nutrition. Across almost all stages of a woman’s life, from adolescence to reproductive years, pregnancy, postpartum, and menopause, experts agreed on individualized support rather than universal supplementation. This is coherent with the modern view of medicine: precision medicine is also reflected in dietary patterns of nutrition recommendations, with particular focus on dietary habits, body mass index, body composition, comorbidities, and inflammatory or metabolic profiles. The panelists’ perspectives align with evidence suggesting that individualized nutritional interventions may enhance reproductive outcomes, improve metabolic control in PCOS and gestational diabetes, modulate inflammatory pathways in endometriosis, and contribute to long-term disease prevention [45,46].

### 4.1. Clinical Evidence Supporting Delphi Recommendations

Available clinical evidence supporting key nutritional interventions across the female life course was reviewed to contextualize the Delphi consensus statements. A large body of evidence supports iron and folate supplementation during pregnancy to prevent maternal anemia, low birth weight, and preterm birth, as reflected in WHO antenatal care recommendations [9,31,33,34]. However, dietary changes are important aspects in PCOS management: evidence shows that they help to improve metabolic and reproductive outcomes [43]. Moreover, nutritional improvements which increase peak bone mass during early adulthood are recognized as important measures against osteoporosis and fracture risk, particularly with adequate calcium and vitamin D intake. In menopause, evidence agrees with the role of adequate calcium and vitamin D intake in maintaining bone health; additionally, diet interventions and weight management contribute to reducing cardiometabolic risk [26,30].

On the other hand, evidence investigating universal vitamin D supplementation during pregnancy or the use of antioxidant or complementary supplements in menopause shows heterogeneous results. These areas of uncertainty are consistent with the lack of consensus among panelists; they underscore the need of high-quality clinical studies to guide future guideline development [26,30].

### 4.2. Pre- and Periconceptional Nutrition

The importance of the periconceptional window [47] is well-established; it is recognized as the main period influencing fertility, early embryonic development, and pregnancy outcomes. Aligning with recent literature [28], panelists agreed on the need for personalized supplementation, especially regarding folates, which remain essential for neural tube defect prevention [34] and homocysteine regulation. The evaluation of risk factors, such as hyperhomocysteinemia, could lead to prescribing different folate forms, with the active form preferred in specific cases. This is coherent with increasing international literature [33] that focuses particularly on interindividual metabolic variability and the potential benefits of specific folate strategies. It has been reported that 5-Metyltetrahydrofolate supplementation, in the presence of MTHFR polymorphism [48], can ameliorate fertility in both female and male counterparts [48]. One-Carbon metabolism is crucial for the production and maturation of gametes, and early cell division of the embryo [49]. Despite a strong biological plausibility, evidence on the role of Folate supplementation on ART outcome is sparse, probably explaining the panelists’ disagreement on the preferred dosage [49,50]. The consensus also emphasizes the need for a particular nutritional assessment in women undergoing ART. Evidence consistently shows the influence of micronutrient adequacy, oxidative balance, and metabolic health on ovarian response and implantation potential [51]. In this context, the experts’ recommendations support the need for structured nutritional protocols in reproductive medicine [33,34,47].

### 4.3. Nutrition During Pregnancy

Important physiological changes characterize the development of pregnancy, with nutritional requirements that increase to support fetal development, placental function, and maternal health. The absence of consensus on whether diet alone is adequate in the second and third trimesters reflects a well-documented challenge: although a balanced diet is theoretically sufficient, common dietary patterns often do not provide optimal amounts of key micronutrients.

Particular attention is on the docosahexaenoic acid (DHA). A strong consensus on its supplementation defines a crucial finding. DHA is well-known [52] for its roles in fetal vision maturation, membrane fluidity, fetal neurodevelopment, and inflammatory modulation. Panelists agreed on a universal supplementation (around 200–300 mg/day) with individualized adjustments. This is consistent with the existing scientific literature. Cetin et al. [53] well defined the association between fatty acids and reduced risk of cardiovascular and other diseases during pregnancy and lactation. Moreover, other benefits regard positive infant neurodevelopmental outcomes, confirming an average dietary docosahexaenoic acid intake of at least 200 mg/day in this period. Moreover, a Cochrane review emphasized the role of omega 3 supplementation in prevention of preterm birth <37 weeks (risk ratio (RR) 0.89, 95% confidence interval (CI) 0.81 to 0.97; 26 RCTs, 10,304 participants; high-quality evidence) and early preterm birth <34 weeks (RR 0.58, 95% CI 0.44 to 0.77; 9 RCTs, 5204 participants; high-quality evidence) [36].

Instead, vitamin D supplementation remained controversial. Although most experts recommended supplementation at modest dosages, consensus on universal supplementation was not reached. This reflects persistent international uncertainty regarding optimal vitamin D levels in pregnancy [37]. Nonetheless, the unanimous support for its use in gestational diabetes is highly relevant, given the growing evidence linking vitamin D status with glucose metabolism [38], insulin resistance [43], and inflammatory pathways in pregnancy [54].

### 4.4. Postpartum Nutrition

The postpartum period received considerable attention from the expert committee. The consensus for iron supplementation in non-breastfeeding women reflects the high prevalence of postpartum iron deficiency due to pregnancy-related depletion and peripartum blood loss. Instead, the lack of consensus on breastfeeding women emphasizes the complexity of postpartum nutritional physiology, the heterogeneity of maternal dietary intake, and the variability of nutrient transmission through breast milk. The close-consensus for folate, B-group vitamins, and iron in breastfeeding women suggests a perceived clinical need but insufficient evidence for universal recommendations. This underlines an important research gap: few high-quality studies studied optimal postpartum micronutrient strategies, although clear associations exist between maternal nutrition, lactation, mood disorders, and fatigue. This is also consistent with the international consensus about nutrition in postpartum lactation [28] underlining the importance of supplementing lactation with vitamin D, iron, DHA and calcium.

The lack of consensus observed for some nutritional recommendations during breastfeeding likely reflects important gaps in the current literature. Although breastfeeding substantially increases maternal nutritional demands, high-quality evidence guiding optimal micronutrient supplementation during lactation remains limited for several nutrients. Existing studies often show heterogeneity in supplementation protocols, maternal nutritional status, and measured outcomes, making it difficult to establish clear and universally applicable recommendations [10,39]. In addition, many available studies focus primarily on infant outcomes rather than maternal nutritional status. These limitations may explain the uncertainty among panelists and highlight the need for well-designed clinical trials evaluating targeted nutritional interventions during the postpartum and lactation period.

### 4.5. Nutrition in Non-Pregnant Reproductive-Age Women

The strong consensus on iron supplementation, as well as previous screening for adolescents with heavy menstrual bleeding, is coherent with public health data showing high rates of iron deficiency in this population [39], even in the absence of anemia. It is essential to prevent fatigue, impaired neurocognitive performance, and reduced academic and physical functioning through early detection. The close consensus on early vitamin D and calcium supplementation by age 30 for bone health reflects the recognition that peak bone mass is achieved by this age. Hopping in bone health during young adulthood may reduce the long-term risk of osteoporosis. Importantly, the panel did **not** support menstrual phase-based cyclical nutritional regimens. Although there is increasing interest in these approaches, evidence remains uncertain and inconsistent, and the experts define the need for caution before translating emerging trends into clinical practice.

### 4.6. Nutrition in Gynecologic Conditions

The increasing number of evidence supporting diet as a fundamental component of symptom management for PCOS and endometriosis patients is aligned with the strong consensus of this Delphi panel supporting diet as a fundamental component of these patients’ management [55]. In PCOS, interventions for insulin resistance, weight reduction, anti-inflammatory pathways, and carbohydrate quality are well-supported. The close-consensus around inositol supplementation reflects its widely recognized role in improving ovulatory function, insulin sensitivity, and metabolic parameters. In endometriosis, increasing interest in anti-inflammatory dietary patterns, including reductions in refined sugars, red meat, and trans fats, and increased consumption of fiber, omega-3 fatty acids, vegetables, and antioxidants, is in line with emerging studies linking diet to pain modulation and inflammatory burden [56]. The panelists’ suggestion of nutritionists’ involvement reflects the complexity of managing endometriosis symptoms through comprehensive lifestyle strategies. For severe premenstrual syndrome (PMS), the absence of consensus likely reflects limited and heterogeneous evidence. Although some studies suggest potential benefits of micronutrients and anti-inflammatory dietary approaches, current data remain insufficient to justify systematic recommendations.

### 4.7. Menopause

Finally, panelists agreed on macronutrient and caloric adjustments in menopause patients to control body composition, insulin sensitivity, and metabolic pathways. Calcium and vitamin D supplementation were the only interventions reaching consensus: this is consistent with their well-known role in lowering the rate of progression of postmenopausal bone loss. The lack of agreement regarding omega-3 fatty acids, antioxidants, or magnesium underscores inconsistent findings in clinical trials and the need for more robust evidence.

### 4.8. Broader Implications

Overall, this Delphi process reveals that experts favor a life-course model of nutritional care in women’s health, recognizing the cumulative impact of early nutritional status on long-term health trajectories. The results emphasize the need for systematic integration of nutritional counseling into routine gynecologic care, greater collaboration between gynecologists and nutrition specialists, and policy-level efforts to move beyond a pregnancy-centered approach to women’s nutrition. A major practical consideration that has emerged from this consensus is the interplay between the personalization of nutritional strategies and the practicability of routine assessment of biomarkers. Although the panel strongly supported the personalization of supplementation based on nutritional status, the practicability of routine laboratory assessment of certain micronutrients might be limited in some systems because of logistical and economic considerations. In such situations, the need for practical considerations might arise. For example, the universality of supplementation strategies at the outset might be continued for certain micronutrients that have a well-established safety profile and high risk of deficiency, such as folate in the pre-conception period and iron supplementation during pregnancy in high-risk groups. On the other hand, the personalization of supplementation based on the assessment of biomarkers might be continued in situations in which excessive supplementation has potential risks and in which the prevalence of deficiency is highly variable.

## 5. Strengths and Limitations

Several major strengths characterize this study. Firstly, experts of the Delphi group were qualified medical clinicians and researchers with expertise in different areas of gynecology and obstetrics, as well as in women’s health and nutrition. The use of the Delphi technique enabled experts to reflect on their responses through a series of follow-up evaluations, in which anonymity helped mitigate hierarchical issues related to consensus development. The unique aspect of this study is its focus on nutritional requirements from adolescence to post-menopause. The breadth of topics explored, combined with a high response rate, provides strengths, robustness, and internal validity to this study. An additional strength is the integration of qualitative comments with quantitative ratings, which enriched the interpretation of consensus levels.

However, there are some limitations to be mentioned. While it would be acceptable, from a methodological standpoint, to include 13 experts in a Delphi study, it would be difficult to generalize the results. Selecting only Italian experts would limit the generalizability of these results to other healthcare systems and cultural contexts, given nutritional differences and medical approaches. Furthermore, although the panel consisted of clinicians with expertise in women’s health and nutrition, it primarily included specialists in gynecology and obstetrics and did not involve clinical nutritionists, dietitians, or endocrinologists. Considering that the focus of the study is nutritional strategies across the female life course, the absence of a broader multidisciplinary panel represents a limitation. Future consensus initiatives should ideally include experts in clinical nutrition, dietetics, endocrinology, and metabolic medicine in order to provide a more comprehensive multidisciplinary perspective and to further validate these findings. The analysis highlighted that there are still several areas of non-consensus, reflecting the need for more robust research in many fields. Moreover, the study did not involve patient perceptions; it would be interesting to collect data from patients related to the acceptability and feasibility of nutritional recommendations. Another limitation relates to the survey design. The questionnaire did not include a “do not know” response option, which may have limited the ability of panelists to express uncertainty regarding specific statements. As noted in some qualitative comments, this may have led experts to select a neutral or alternative response even when their position reflected uncertainty rather than disagreement. Future Delphi surveys in this field should consider including a “do not know” option to better capture areas of uncertainty among experts. Finally, not being able to determine specific criteria related to nutritional biomarkers would make it difficult to standardize nutritional supplementation according to different situations.

## 6. Conclusions

The present study is a Delphi consensus which aimed to emphasize the crucial role of nutrition in every stage of a woman’s life. The panelists underline the necessity of unified, evidence-informed guidelines to support clinicians in daily practice. While experts strongly recommend personalized nutritional strategies, key gaps remain, in particular, regarding vitamin D in physiological pregnancy, micronutrient needs during breastfeeding, and dietary interventions for premenstrual disorders.

Findings of this study provide an essential urge for future guideline development. It tried to identify priority areas for research, including standardized protocols for nutritional assessment. The evaluation of biomarkers and the possibility of randomized trials addressing nutritional interventions across different life stages are the successive strategies. Integrating structured nutritional counseling into gynecologic and obstetric care may have profound implications for women’s health, well-being, and long-term disease prevention.

This consensus represents a significant step toward a future life-course approach to women’s nutrition and suggests that recommendations, supported by growing evidence and expert agreement, can meaningfully improve clinical practice and patient outcomes.

## Figures and Tables

**Table 1:** Composition of the Delphi Consensus Working Group.

Expert	Institution	Geographic Area
Irene Cetin	University of Milan	Northern Italy
Enrico Mario Ferrazzi	Fondazione IRCCS Ca’ Granda Ospedale Policlinico di Milano	Northern Italy
Fabio Parazzini	University of Milan	Northern Italy
Francesca Parisi	University of Milan	Northern Italy
Rossella Elena Nappi	University of Pavia	Northern Italy
Daniele Di Mascio	Sapienza University of Rome	Central Italy
Tullio Ghi	Fondazione Policlinico Universitario A. Gemelli IRCCS	Central Italy
Giuseppe Rizzo	Sapienza University of Rome	Central Italy
Luigi Nappi	University of Foggia	Southern Italy
Pasquale De Franciscis	University of Campania “Luigi Vanvitelli”	Southern Italy
Maurizio Guida	University Federico II	Southern Italy
Laura Sarno	University Federico II	Southern Italy
Gabriele Saccone	University Federico II	Southern Italy

**Table 2:** Comparison of Delphi consensus statements with international guideline recommendations and supporting evidence.

Delphi Statement	Delphi Result	Guideline Comparison	Evidence Summary
Personalized supplementation based on individual deficiencies (preconception)	Consensus	Consistent with WHO emphasis on optimizing maternal nutritional status [31]	Maternal micronutrient status before conception influences placental development and pregnancy outcomes [5,32]
Folate supplementation in ART patients	Consensus	WHO recommends folic acid supplementation in the periconception period [31]	Folate supplementation reduces neural tube defects and supports early placental development [9,31,33,34]
Diet alone sufficient in 2nd–3rd trimester	No consensus	WHO recommends routine iron–folate supplementation [31]	Dietary intake alone often fails to meet micronutrient requirements in pregnancy [10,35]
Personalized supplementation in pregnancy	Consensus	Consistent with WHO iron–folate recommendations [31]	Iron supplementation reduces maternal anemia and adverse pregnancy outcomes [10,35]
DHA supplementation in pregnancy	Consensus	Not specifically addressed by WHO	Omega-3 supplementation may reduce preterm birth risk [13,36]
Universal vitamin D supplementation in pregnancy	No consensus	WHO does not recommend routine vitamin D supplementation [31]	Evidence remains heterogeneous [37,38]
Iron supplementation postpartum	Consensus	Consistent with WHO postpartum anemia management [31]	Improves maternal recovery and anemia [10,39]
Iron supplementation for adolescents with heavy menstrual bleeding	Consensus	ACOG recommends evaluation and iron therapy when deficiency is present [40]	Iron deficiency common in adolescents with heavy menstrual bleeding [39]
Early nutritional strategies for bone health before age 30	Near consensus	Consistent with WHO bone health recommendations [41]	Calcium and vitamin D intake influence peak bone mass acquisition [14,30]
PCOS personalized nutrition	Consensus	Consistent with PCOS guideline recommending individualized healthy diet [42]	Lifestyle interventions improve metabolic and reproductive outcomes [43]
Inositol supplementation in PCOS	Consensus	PCOS guideline: may be considered with limited evidence [42]	May improve insulin sensitivity but clinical benefits remain limited [17]
Menopause diet and body composition	Consensus	Consistent with European menopause guidelines recommending lifestyle management [44]	Menopause associated with increased cardiometabolic risk [26,30]
Calcium and vitamin D in menopause	Consensus	Consistent with osteoporosis prevention guidelines [44]	Supports bone mineral density and fracture prevention [30]

## Data Availability

All data generated or analyzed during this study are available upon reasonable request.

## Supplementary Materials

The following supporting information can be downloaded at: https://www.mdpi.com/article/10.3390/nu18071053/s1, Supplementary Materials S1: Round 1 Delphi questionnaire; Supplementary Materials S2: Round 2 Delphi questionnaire.

## Abbreviations

The following abbreviations are used in this manuscript:

AGUI	Italian Association of University Gynecologists and Obstetricians
ART	Assisted Reproductive Technology
BMI	Body Mass Index
CI	Confidence Interval
DHA	Docosahexaenoic Acid
EFSA	European Food Safety Authority
GDM	Gestational Diabetes Mellitus
GDPR	General Data Protection Regulation
ICMJE	International Committee of Medical Journal Editors
IQR	Interquartile Range
MTHFR	Methylenetetrahydrofolate Reductase
PCOS	Polycystic Ovary Syndrome
PMS	Premenstrual Syndrome
RCTs	Randomized Controlled Trials
RR	Risk Ratio
WHO	World Health Organization
