# Supplementary material for: Italian Expert Consensus on Women’s Nutrition Across the Life Course: A Modified Delphi Study
Source: Nutrients. 2026 Mar 26;18(7):1053. doi: 10.3390/nu18071053 (PMC13074548; doi:10.3390/nu18071053)
Supplement: Supplementary file 1 [file nutrients-18-01053-s001.zip › Supp mat 1st round questions.pdf]

The consensus questions were translated from Italian to English using an automatic translator. The content was subsequently reviewed by the authors.

For each of the following statements, we kindly ask you to indicate your level of agreement using a 10-point Likert scale (0–9). Values from 0 to 5 indicate increasing levels of disagreement, the value 6 corresponds to agreement, while values from 7 to 9 represent strong agreement. Each response is accompanied by the option to provide an open-ended comment: we invite you to use this space to offer motivations, clarifications, or points for reflection, especially in cases where consensus is not reached. The process will follow the Delphi methodology and will be carried out over multiple rounds, with the aim of progressively refining positions and reaching a shared agreement.

PRE/PERICONCEPTIONAL PERIOD

1) Is there information and awareness about the importance of nutrition during the different stages of a woman's life?

n*	Somewhat to strongly disagree (%)	Neutral (%)	Somewhat to strongly agree (%)
13	8 (61,5%)	2 (15,4%)	3 (23,1%)

Comments:

- Little attention from both healthcare professionals and patients; increasing obesity rates with inadequate nutritional patterns.
- Rarely included in specialist counselling during the preconception phase.

2) In the pre/periconceptional period, in the absence of pathological conditions:
- A balanced diet is sufficient

n*	Somewhat to strongly disagree (%)	Neutral (%)	Somewhat to strongly agree (%)
13	7 (53,8%)	1 (7,7%)	5 (38,5%)

Comments:

- There is a need for folic acid supplementation
- It is advisable to check certain markers of vitamin and micronutrient absorption, such as folate levels, Vitamin D3, ferritin
- Folic acid and other vitamins are also necessary

2) In the pre/periconceptional period, in the absence of pathological conditions:
- A specific nutritional intervention is necessary

n*	Somewhat to strongly disagree (%)	Neutral (%)	Somewhat to strongly agree (%)
13	9 (69,2%)	0 (0%)	4 (30,8%)

Comments:

- Vitamin B9 pathway in subjects with homocysteine values above 12; folic acid is also appropriate in subjects with homocysteine < 8
- Only if overweight or obese
- Most of the time it is not necessary

2) In the pre/periconceptional period, in the absence of pathological conditions:<br>- A generic supplementation is sufficient			
**n***	**Somewhat to strongly disagree (%)**	**Neutral (%)**	**Somewhat to strongly agree (%)**
13	8 (61,5%)	2 (15,4%)	3 (23,1%)

Comments:

- It is important to work on the adequacy of the nutritional model, which differs among Caucasian, Asian, and South American populations, and to be familiar with national dietary recommendations and multivitamin compositions
- It is important that it contains at least the minimum recommended dose of folic acid
- Mainly folic acid
- Specific supplementation is necessary, such as folic acid

2.2) In the pre/periconceptional period, in the absence of pathological conditions:<br>- A personalized supplementation is necessary based on each patient's dietary deficiencies			
**n***	**Somewhat to strongly disagree (%)**	**Neutral (%)**	**Somewhat to strongly agree (%)**
13	1 (7,7%)	1 (7,7%)	11 (84,6%)

Comments:

- Vitamin D, for example, or iodine
- Personalization is preferable, but not always possible

3) How important is folate supplementation in patients undergoing ART (Assisted Reproductive Techniques)?			
**n***	**Somewhat to strongly disagree (%)**	**Neutral (%)**	**Somewhat to strongly agree (%)**
13	1 (7,7%)	0 (0%)	12 (92,3%)

Comments:

- Recent evidence suggests improved ART success when associated with vitamin B9
- It is essential to reduce homocysteine and inflammation
- Not more important than in all women trying to conceive

4) At what dosage would you supplement folates in patients undergoing ART?			
**n***	**400 mcg (%)**	**5 mg (%)**	**>5 mg (%)**
13	8 (61,5%)	2 (15,4%)	3 (23,1%)

Comments:

- Supplementation with folates in their active form
- It is important not to prescribe folic acid to patients with hyperhomocysteinemia, but rather the active form
- Depends on the presence of associated risk factors

- Considering that assessing polymorphisms associated with hyperhomocysteinemia is not mandatory, more than the dosage, I would focus on the type of folate used (active form rather than folic acid)

PREGNANCY

5) In the second and third trimester of pregnancy, in the absence of pathological conditions:

- A balanced diet is sufficient

n*	Somewhat to strongly disagree (%)	Neutral (%)	Somewhat to strongly agree (%)
13	7 (53,8%)	2 (15,4%)	4 (30,8%)

Comments:

- Supplementation with vitamin B9 should be continued throughout the entire pregnancy; vitamin D should be supplemented in subjects with deficiency or reduced/absent sun exposure; iron in the form of protected ferrous sulfate should be given to subjects with abnormal ferritin levels; omega-3 in women with limited intake of adequate dietary sources
- Supplementation is necessary

5.2) In the second and third trimester of pregnancy, in the absence of pathological conditions:

- A specific nutritional intervention is necessary

n*	Somewhat to strongly disagree (%)	Neutral (%)	Somewhat to strongly agree (%)
13	7 (53,8%)	2 (15,4%)	4 (30,8%)

Comments:

- Treatment of gastric conditions (viral infections, gastritis) and intestinal disorders (constipation, colicky evacuation pain, post-meal bloating) is recommended, if needed with referral to nutrition specialists, and always taking into account the dietary pyramids of major ethnic groups
- It depends on the patient’s clinical characteristics

5.3) In the second and third trimester of pregnancy, in the absence of pathological conditions:

- Generic supplementation is sufficient

n*	Somewhat to strongly disagree (%)	Neutral (%)	Somewhat to strongly agree (%)
13	7 (53,8%)	2 (15,4%)	4 (30,8%)

5) In the second and third trimester of pregnancy, in the absence of pathological conditions:

- Personalized supplementation is necessary based on dietary deficiencies of each patient

n*	Somewhat to strongly disagree (%)	Neutral (%)	Somewhat to strongly agree (%)

13	1 (7,7%)	2 (15,4%)	10 (76,9%)

6) To what extent do you consider nutritional personalization based on individual factors (pre-pregnancy BMI, inflammatory profile, microbiota, etc.) useful, even in physiological pregnancies?

n*	Slightly not to not at all important (%)	Neutral (%)	Slightly to extremely important (%)
13	1 (7,7%)	0 (0%)	12 (92,3%)

Comments:

- Higher folic acid doses in women with severe obesity

7) Is it necessary to specifically adapt the diet in the different trimesters of pregnancy, even in the absence of pathologies?

n*	Slightly not to not at all important (%)	Neutral (%)	Slightly to extremely important (%)
13	3 (23,1%)	2 (15,4%)	8 (61,5%)

Comments:

- Iron, calcium

8) To what extent is universal DHA supplementation in pregnancy justifiable?

n*	Slightly not to not at all important (%)	Neutral (%)	Slightly to extremely important (%)
13	3 (23,1%)	3 (23,1%)	7 (53,8%)

Comments:

- It depends on fish intake in the diet
- For subjects who do not consume adequate dietary sources
- Supplementation should be adjusted based on DHA intake (at least 150 mg/day) and specific risk factors (e.g., risk of preterm birth)

8.2) From which trimester should supplementation ideally begin?

n*	First trimester (%)	Second trimester (%)	Third trimester (%)
13	8 (61,5%)	5 (38,5%)	(0%)

8.3) How concerned should one be about potential side effects?

n*	Slightly not to not at all important (%)	Neutral (%)	Slightly to extremely important (%)

13	7 (53,8%)	0 (0%)	6 (46,2%)

Comments:

- Gastritis/heartburn, dark stools in patients supplemented with non-protected ferrous sulfate
- Possible issues with digestion and reflux

9) To what extent is universal vitamin D supplementation in pregnancy justified?

n*	Slightly not to not at all important (%)	Neutral (%)	Slightly to extremely important (%)
13	7 (53,8%)	2 (15,4%)	5 (38,5%)

Comments:

- In general, it is useful but its role has not yet been clearly demonstrated

9.2) From which trimester?

n*	First trimester (%)	Second trimester (%)	Third trimester (%)
13	10 (76,9%)	2 (15,4%)	1 (7,7%)

9.3) At what dosage?

n*	1000 UI(%)	2000 UI (%)	4000 UI (%)
13	3 (23,1%)	9 (69,2%)	1 (7,7%)

9.4) How concerned should one be about potential side effects?

n*	Slightly not to not at all important (%)	Neutral (%)	Slightly to extremely important (%)
13	8 (61,5%)	0 (0%)	5 (38,5%)

9.5) How important do you consider serum vitamin D measurement?

n*	Slightly not to not at all important (%)	Neutral (%)	Slightly to extremely important (%)
13	8 (61,5%)	2 (15,4%)	3 (23,1%)

Comments:

- Given that vitamin D deficiency is very common, it may not be worth the cost of serum testing

10) To what extent is vitamin D supplementation important in pregnancy complicated by obesity?

n*	Slightly not to not at all important (%)	Neutral (%)	Slightly to extremely important (%)

13	3 (23,1%)	0 (0%)	10 (76,9%)

11) To what extent is vitamin D supplementation important in pregnancy complicated by gestational diabetes mellitus?

n*	Slightly not to not at all important (%)	Neutral (%)	Slightly to extremely important (%)
13	4 (30,8%)	1 (7,7%)	8 (61,5%)

12) To what extent is vitamin D supplementation important in a pregnancy where the patient experienced gestational hypertension in a previous pregnancy?

n*	Slightly not to not at all important (%)	Neutral (%)	Slightly to extremely important (%)
13	1 (7,7%)	4 (30,8%)	8 (61,5%)

Comments:

- Evidence is contradictory, but supplementation is reasonable

13) Would you agree with the fortification of foods (flour, bread) with folic acid?

n*	Slightly not to not at all important (%)	Neutral (%)	Slightly to extremely important (%)
13	7 (53,8%)	1 (7,7%)	5 (38,5%)

Comments:

- Simple food fortification is not sufficient to reach the recommended pre-pregnancy levels
- Yes, but first it is necessary to teach people how to eat properly, including cooking methods, etc.

14) How important is folate supplementation in the second trimester of pregnancy in patient without anemia?

n*	Slightly not to not at all important (%)	Neutral (%)	Slightly to extremely important (%)
13	2 (15,4%)	4 (30,8%)	7 (53,8%)

Comments:

- The B9 pathway is involved in the synthesis of purines and thymine, the protection of methyl groups (epigenetics), reduction of homocysteine levels, and vascular risk
- It is useful due to its anti-inflammatory role

15) How important is folate supplementation in the third trimester of pregnancy in the absence of anemia?

n*	Slightly not to not at all important (%)	Neutral (%)	Slightly to extremely important (%)
13	3 (23,1%)	4 (30,8%)	6 (46,1%)

PUERPERIUM

16) Considering multivitamin supplementation in the postpartum period, which nutrients would you recommend for a healthy patient who is not breastfeeding?

n*	Slightly not to not at all important (%)	Neutral (%)	Slightly to extremely important (%)
13			
Folates	4 (30,8%)	5 (38,5%)	4 (30,8%)
DHA	11 (84,6%)	0 (0%)	2 (15,4%)
Selenium	11 (84,6%)	2 (15,4%)	0 (0%)
Calcium	5 (38,5%)	2 (15,4%)	6 (46,1%)
Magnesium	6 (46,1%)	3 (23,1%)	4 (30,8%)
Iodine	9 (69,2%)	2 (15,4%)	2 (15,4%)
Vitamin K	11 (84,6%)	2 (15,4%)	0 (0%)
Vitamin D	5 (38,5%)	2 (15,4%)	6 (46,1%)
B Group Vitamins	7 (53,8%)	0 (0%)	6 (46,1%)
Iron	1 (7,7%)	4 (30,8%)	8 (61,5%)

16.2) Considering multivitamin supplementation in the postpartum period, for how long which nutrients would you recommend for a healthy patient who is not breastfeeding?

n*	1 month (%)	3 months (%)	1 year (%)
13			
Folates	7 (53,8%)	5 (38,5%)	1 (7,7%)
DHA	9 (69,2%)	4 (30,8%)	0 (0%)
Selenium	8 (61,5%)	5 (38,5%)	0 (0%)
Calcium	6 (46,1%)	5 (38,5%)	2 (15,4%)
Magnesium	7 (53,8%)	5 (38,5%)	1 (7,7%)
Iodine	9 (69,2%)	4 (30,8%)	0 (0%)
Vitamin K	10 (76,9%)	3 (23,1%)	0 (0%)
Vitamin D	6 (46,1%)	5 (38,5%)	1 (7,7%)
B Group Vitamins	6 (46,1%)	7 (53,8%)	0 (0%)
Iron	6 (46,1%)	6 (46,1%)	1 (7,7%)

Comments:

- Selenium
  - Limited data available in the literature
- Calcium
  - Also through calcium-rich mineral waters
  - Depends on lifestyle and breastfeeding
  - Pregnancy and breastfeeding not supported by supplementation are risk factors for osteopenia in menopause
- Magnesium
  - “Don’t know” option missing
- Iodine
  - To be evaluated based on thyroid markers
  - “Don’t know” option missing
- Vitamin K
  - “Don’t know” option missing
- B-group vitamins
  - “Don’t know” option missing
- Iron
  - Should be continued after breastfeeding
  - Depends on haemoglobin at delivery
  - Depends on Hb values

17) Considering multivitamin supplementation in the postpartum period, which nutrients would you recommend for a healthy patient who is breastfeeding?

**n***	**Slightly not to not at all important (%)**	**Neutral (%)**	**Slightly to extremely important (%)**
13			
Folates	1 (7,7%)	3 (23,1%)	9 (69,2%)
DHA	6 (46,1%)	4 (30,8%)	3 (23,1%)
Selenium	9 (69,2%)	2 (15,4%)	2 (15,4%)
Calcium	3 (23,1%)	3 (23,1%)	7 (53,8%)
Magnesium	8 (61,5%)	2 (15,4%)	3 (23,1%)

Iodine	7 (53,8%)	1 (7,7%)	5 (38,5%)
Vitamin K	10 (76,9%)	1 (7,7%)	2 (15,4%)
Vitamin D	5 (38,5%)	1 (7,7%)	7 (53,8%)
B Group Vitamins	3 (23,1%)	1 (7,7%)	9 (69,2%)
Iron	3 (23,1%)	1 (7,7%)	9 (69,2%)

17.2) Considering multivitamin supplementation in the postpartum period, for how long which nutrients would you recommend for a healthy patient who is breastfeeding?

n*	1 month (%)	3 months (%)	1 year (%)
13			
Folates	2 (15,4%)	8 (61,5%)	3 (23,1%)
DHA	2 (15,4%)	7 (53,8%)	4 (30,8%)
Selenium	5 (38,5%)	8 (61,5%)	0 (0%)
Calcium	0 (0%)	8 (61,5%)	5 (38,5%)
Magnesium	4 (30,8%)	8 (61,5%)	1 (7,7%)
Iodine	3 (23,1%)	10 (76,9%)	0 (0%)
Vitamin K	6 (46,1%)	7 (53,8%)	0 (0%)
Vitamin D	2 (15,4%)	6 (46,1%)	5 (38,5%)
B Group Vitamins	2 (15,4%)	9 (69,2%)	2 (15,4%)
Iron	2 (15,4%)	10 (76,9%)	1 (7,7%)

Comments:

- Folates
  - During breastfeeding
  - Ideally throughout exclusive breastfeeding
- DHA
  - During breastfeeding
- Selenium
  - “Don’t know” option missing
- Calcium
  - During breastfeeding
- Magnesium
  - “Don’t know” option missing
- Iodine
  - Depending on thyroid function markers

- Vitamin K
    - “Don’t know” option missing
- Vitamin D
    - Depends on diet, season, ethnicity
- B-group vitamins
    - “Don’t know” option missing
- Iron
    - In protected form
    - If iron-deficiency anemia

ROUTINE SUPPLEMENTATION IN A WOMAN’S LIFE

18) How advisable is iron supplementation or increased intake of iron-rich foods in adolescents with heavy menstrual bleeding, in the absence of anemia?

**n***	**Slightly not to not at all important (%)**	**Neutral (%)**	**Slightly to extremely important (%)**
13	3 (23,1%)	2 (15,4%)	8 (61,5%)

Comments:

- Based on complete blood count results

19) Is it appropriate to intervene early (before age 30) on diet for women’s bone health?

**n***	**Slightly not to not at all important (%)**	**Neutral (%)**	**Slightly to extremely important (%)**
13	5 (38,5%)	0 (0%)	8 (61,5%)

Comments:

- Calcium-rich mineral waters, aged cheeses, cruciferous vegetables; avoid hydration exclusively with low-mineral-content waters

20) How acceptable is the “cyclical” nutritional approach, i.e., modulated according to hormonal phases of the menstrual cycle (e.g., follicular vs luteal phase)?

**n***	**Slightly not to not at all important (%)**	**Neutral (%)**	**Slightly to extremely important (%)**
13	8 (61,5%)	1 (7,7%)	4 (30,8%)

Comments:

- Not demonstrated

ROUTINE SUPPLEMENTATION IN WOMEN WITH PATHOLOGICAL CONDITIONS

21) How useful is nutritional personalization in the management of endometriosis?

n*	Slightly not to not at all important (%)	Neutral (%)	Slightly to extremely important (%)
13	5 (38,5%)	0 (0%)	8 (61,5%)

Comments:

- Excluding inflammatory bowel diseases; assessing potential inflammatory dietary antigens such as gluten, lactose (in lactase non-persistent individuals), yeasts

22) How useful is nutritional personalization in the management of polycystic ovary syndrome (PCOS)?

n*	Slightly not to not at all important (%)	Neutral (%)	Slightly to extremely important (%)
13	1 (7,7%)	1 (7,7%)	11 (84,6%)

Comments:

- Weight control
- Reduction or elimination of sucrose, reduction of high–glycaemic index carbohydrates, increased fibre and healthy protein intake, protein-based breakfast

23) How useful is nutritional personalization in the management of severe premenstrual syndrome (PMS)?

n*	Slightly not to not at all important (%)	Neutral (%)	Slightly to extremely important (%)
13	3 (23,1%)	1 (7,7%)	9 (69,2%)

Comments:

- Reduce intestinal inflammation induced by food and inflammatory cytokine production

24) How important do you consider supplementation with inositols in the following cases:

n*	Slightly not to not at all important (%)	Neutral (%)	Slightly to extremely important (%)
13			
PCOS	1 (7,7%)	0 (0%)	12 (92,3%)
Idiopathic infertility	8 (61,5%)	4 (30,8%)	1 (7,7%)

Gestational diabetes	0 (0%)	1 (7,7%)	12 (92,3%)

Comments:

- PCOS
  - Depends on metabolic phenotype
  - To be associated with dietary control, physical activity, and glycaemic profile monitoring
- Idiopathic infertility
  - No evidence
- Gestational diabetes
  - In pregnant patients with metabolic PCOS

MENOPAUSE

25) To what extent do you consider it appropriate to introduce phytonutrients (e.g., isoflavones, flavonoids) into the diet of women in perimenopause, even in the absence of symptoms?

n*	Slightly not to not at all important (%)	Neutral (%)	Slightly to extremely important (%)
13	7 (53,8%)	1 (7,7%)	5 (38,5%)

Comments:

- There does not seem to be sufficient evidence

26) Is it justified to propose a modification of caloric intake and macronutrients in menopausal women to account for changes in body fat distribution?

n*	Slightly not to not at all important (%)	Neutral (%)	Slightly to extremely important (%)
13	2 (15,4%)	0 (0%)	11 (84,6%)

27) To what extent do you consider a diet rich in omega-3, antioxidants, and micronutrients helpful in preventing cognitive decline during menopause?

n*	Slightly not to not at all important (%)	Neutral (%)	Slightly to extremely important (%)

13	2 (15,4%)	3 (23,1%)	8 (61,5%)

28) Which of the following nutrients do you consider important to supplement in perimenopausal patients?

n*	**Slightly not to not at all important (%)**	**Neutral (%)**	**Slightly to extremely important (%)**
13			
Folates	4 (30,8%)	4 (30,8%)	5 (38,5%)
DHA	7 (53,8%)	0 (0%)	6 (46,2%)
Selenium	8 (61,5%)	1 (7,7%)	4 (30,8%)
Calcium	0 (0%)	1 (7,7%)	12 (92,3)
Magnesium	4 (30,8%)	1 (7,7%)	8 (61,5%)
Iodine	6 (46,2%)	3 (23,1%)	4 (30,8%)
Vitamin K	10 (76,9%)	1 (7,7%)	2 (15,4%)
Vitamin D	1 (7,7%)	2 (15,4%)	10 (76,9%)
B Group Vitamins	3 (23,1%)	5 (38,5%)	5 (38,5%)
Iron	7 (53,8%)	2 (15,4%)	4 (30,8%)

Comments:

- Folates
    - Folates are essential throughout all life stages
- DHA
    - Preferably obtained from dietary sources
- Selenium
    - "Don't know" option missing
- Calcium
    - Mainly from dietary sources
- Magnesium
    - "Don't know" option missing
- Iodine
    - "Don't know" option missing
- Vitamin K
    - "Don't know" option missing
- Vitamin D

- In case of deficiency

- Iron
  - If needed
  - In deficiency, monitoring for gastrointestinal side effects

29) Which of the following nutrients do you consider important to supplement in women using combined estrogen–progestin contraception?

n*	Slightly not to not at all important (%)	Neutral (%)	Slightly to extremely important (%)
13			
Folates	6 (46,2%)	2 (15,4%)	5 (38,5%)
DHA	10 (76,9%)	1 (7,7%)	2 (15,4%)
Selenium	12 (92,3)	0 (0%)	1 (7,7%)
Calcium	9 (69,2%)	3 (23,1%)	1 (7,7%)
Magnesium	10 (76,9%)	1 (7,7%)	2 (15,4%)
Iodine	10 (76,9%)	3 (23,1%)	0 (0%)
Vitamin K	11 (84,6%)	2 (15,4%)	0 (0%)
Vitamin D	7 (53,8%)	1 (7,7%)	5 (38,5%)
B Group Vitamins	7 (53,8%)	3 (23,1%)	3 (23,1%)
Iron	7 (53,8%)	5 (38,5%)	1 (7,7%)

30) Would you be in favor of estrogen–progestin contraceptives fortified with folates?

n*	Slightly not to not at all important (%)	Neutral (%)	Slightly to extremely important (%)
13	6 (46,2%)	1 (7,7%)	6 (46,2%)

Comments:

- Previous experiences have not been favorable
- Not very useful unless accompanied by adequate dietary habits
