# Supplementary material for: Italian Expert Consensus on Women’s Nutrition Across the Life Course: A Modified Delphi Study
Source: Nutrients. 2026 Mar 26;18(7):1053. doi: 10.3390/nu18071053 (PMC13074548; doi:10.3390/nu18071053)
Supplement: Supplementary file 1 [file nutrients-18-01053-s001.zip › Supp mat 2nd round questions.pdf]

The consensus questions were translated from Italian to English using an automatic translator. The content was subsequently reviewed by the authors.

For each of the following statements, we kindly ask you to indicate your level of agreement using a 10-point Likert scale (0–9). Values from 0 to 5 indicate increasing levels of disagreement, the value 6 corresponds to agreement, while values from 7 to 9 represent strong agreement. Each response is accompanied by the option to provide an open-ended comment: we invite you to use this space to offer motivations, clarifications, or points for reflection, especially in cases where consensus is not reached. The process will follow the Delphi methodology and will take place over multiple rounds, with the aim of progressively refining positions and converging toward a shared agreement.

1) In the first round, opinions on DHA supplementation during pregnancy were heterogeneous:

- 7 out of 13 respondents (54%) were in favor (Likert 7–9),
- 6 out of 13 (46%) expressed neutral or contrary positions (0–6).

Many comments highlighted that supplementation should be adjusted according to dietary fish intake and specific risk factors (e.g., low DHA intake, risk of preterm birth). In light of these elements, and considering the difficulty—common in many populations—of reaching the recommended intake through diet alone:

Do you consider it justifiable to recommend DHA supplementation during pregnancy for all women, starting from the first trimester, with a standard dosage of 200–300 mg/day, to be adjusted only in cases of high fish consumption or the presence of specific risk factors?

**n***	**Slightly not to not at all important (%)**	**Neutral (%)**	**Slightly to extremely important (%)**
13	2 (15,4%)	1 (7,7%)	10 (76,9%)

Comments:

- Supplementation with high doses of omega-3 should be reserved for specific at-risk populations or those with deficient intake/status. There are no data supporting universal supplementation from the first trimester. However, given the absence of an upper safety limit and observational data suggesting modulation of inflammation and improved oocyte/embryo quality, early supplementation may be reasonable.
- A dosage of 200–300 mg/day is recommended for all pregnant women. Given that only a minority of women consume sufficient fish, supplementation is important.
- Starting from the beginning of the second trimester may be even more appropriate.

2) In the first round, opinions on the clinical relevance of side effects associated with supplementation (e.g., iron, DHA, vitamins) were divided:

- 7 out of 13 respondents (54%) considered them minimally relevant (Likert 0–5),
- 6 out of 13 (46%) considered them more significant (Likert 7–9).

Comments reported mild and manageable symptoms such as gastritis, heartburn, or bowel changes, more frequent with unprotected iron or high-dose supplements. Considering that these effects are generally transient and can be reduced with appropriate formulations (e.g., protected iron, purified DHA, intake with meals):

Do you consider that the side effects associated with DHA supplementation in pregnancy are clinically of little relevance and should not limit the recommendation of appropriate supplementation when indicated?

n*	Slightly not to not at all important (%)	Neutral (%)	Slightly to extremely important (%)
13	3 (23,1%)	0 (0%)	10 (76,9%)

Comments:

- Supplementation—particularly formulations of animal origin—frequently causes side effects in the first trimester, such as worsening of nausea/vomiting.
- It depends on the preparation, but in general DHA-related side effects are mild.

3) In the first round, opinions on universal vitamin D supplementation during pregnancy were divergent:
7 out of 13 participants (53%) expressed disagreement (Likert 0-5),
5 out of 13 (38%) were in favor (Likert 7-9).

However, most participants acknowledged that vitamin D deficiency is highly prevalent and that serum testing is not always necessary for practical or economic reasons. The most frequently suggested dosage was 2000 IU/day, ideally starting from the first trimester. Side effects were considered rare and mild. In light of these considerations, and of the growing evidence on the role of vitamin D in maternal and fetal health (bone, immune, and metabolic function):
Do you consider it justifiable to recommend universal vitamin D supplementation during pregnancy (e.g., 2000 IU/day) from the first trimester, without the need for routine serum testing, except in special conditions or in the presence of a risk of hypercalcemia?

n*	Slightly not to not at all important (%)	Neutral (%)	Slightly to extremely important (%)
13	4 (30,7%)	1 (7,7%)	8 (61,5%)

Comments:
- Supplementation should be calibrated based on risk factors (e.g., extreme BMI), seasonality, lifestyle.
- Dosage should be at least 600 IU/day and up to 2000 IU/day.
- There is no evidence supporting universal supplementation.
- The literature does not support this approach.

4) In the first round, most participants (8 out of 13, 67%) agreed on the importance of vitamin D supplementation in pregnancies complicated by gestational diabetes mellitus (GDM), while 5 out of 13 were more neutral or in disagreement. It was acknowledged that vitamin D may have beneficial effects on glucose metabolism, insulin sensitivity, and reduction of systemic inflammation, with a good safety profile even at dosages of 1000-2000 IU/day. In light of these considerations:
Do you consider it important to systematically recommend vitamin D supplementation (1000-2000 IU/day) in women with gestational diabetes mellitus, regardless of initial serum values, as support for metabolic control and maternal-fetal wellbeing?

n*	Slightly not to not at all important (%)	Neutral (%)	Slightly to extremely important (%)
13	3 (23,1%)	0 (0%)	10 (76,9%)

Comments:

- The literature does not support this.

5) In the first round, most participants (8 out of 13, 67%) agreed on the importance of vitamin D supplementation in women who experienced gestational hypertension in a previous pregnancy.

Some participants noted that the scientific evidence remains contradictory, but supplementation appears feasible and safe, particularly in subjects with deficiency or limited sun exposure. Considering the potential role of vitamin D in maintaining endothelial function, modulating immune responses, and reducing the risk of hypertensive complications:

Do you consider it important to recommend vitamin D supplementation (1000-2000 IU/day) in women with a history of gestational hypertension, even in the absence of documented deficiency, as a preventive measure and support for maternal-fetal wellbeing?

n*	Slightly not to not at all important (%)	Neutral (%)	Slightly to extremely important (%)
13	6 (46,1%)	2 (15,4%)	5 (38,5%)

Comments:

- The literature does not support this.

6) In the first round, most participants (8 out of 13, 62%) agreed on the importance of iron supplementation in the postpartum period even in women who are not breastfeeding, with dosage modulation based on hemoglobin and ferritin values at delivery.

Divergent opinions mainly concerned the duration of supplementation and the need for laboratory monitoring. Considering that postpartum iron deficiency is common, even in the absence of clinically overt anemia, and that supplementation has a good safety profile:

Do you consider it advisable to recommend iron supplementation in the postpartum period for women who are not breastfeeding, continuing for at least 3 months after delivery, and adjusting the dosage based on blood values or symptoms of deficiency?

n*	Slightly not to not at all important (%)	Neutral (%)	Slightly to extremely important (%)
13	0 (0%)	2 (15,4%)	11 (84,6%)

Comments:

- I consider it important, but based on Hb values.

7) In the first round, most participants (9 out of 13, 69%) agreed on the importance of folate supplementation in breastfeeding women, while some suggested adjusting duration to the phase and length of breastfeeding.

It is well known that folate requirements remain high in the postpartum period due to their role in maternal energy metabolism, DNA synthesis, and breast milk composition, with an excellent safety profile. In light of these considerations:

Do you consider it advisable to recommend folate supplementation for all breastfeeding women throughout the entire duration of breastfeeding, regardless of diet or baseline blood values?

n*	Slightly not to not at all important (%)	Neutral (%)	Slightly to extremely important (%)
13	3 (23,1%)	2 (15,4%)	8 (61,5%)

Comments:

- Only in the presence of anemia.

8) In the first round, most participants (9 out of 13, 69%) agreed on the importance of B-vitamin supplementation in breastfeeding women, although some expressed uncertainty or preferred a more selective approach.

B-group vitamins (particularly B6, B12, and B9/folate) play a key role in energy metabolism, nervous system function, and breast milk synthesis. Deficiency may be common postpartum, especially in vegetarians or women with restrictive diets. Considering increased requirements and good tolerability:

Do you consider it advisable to recommend B-vitamin supplementation for all breastfeeding women for at least 3 months, to support maternal wellbeing and the nutritional quality of breast milk, regardless of documented deficiencies?

n*	Slightly not to not at all important (%)	Neutral (%)	Slightly to extremely important (%)
13	5 (38,5%)	0 (0%)	8 (61,5%)

Comments:

- The literature does not support this.

9) In the first round, most participants (9 out of 13, 69%) agreed on the importance of iron supplementation in breastfeeding women, with repeated suggestions to prefer protected formulations for better gastrointestinal tolerance.

The postpartum period is characterized by increased iron needs due to blood loss at delivery and the maintenance of maternal stores during breastfeeding, even without overt anemia. Considering that iron deficiency is common and may contribute to fatigue, reduced cognitive performance, and worsening postpartum mood:

Do you consider it advisable to recommend iron supplementation during breastfeeding for at least 3 months, using well-tolerated formulations (e.g., protected or liposomal iron), to prevent or correct iron deficiency even in the absence of manifest anemia?

n*	Slightly not to not at all important (%)	Neutral (%)	Slightly to extremely important (%)
13	3 (23,1%)	1 (7,7%)	9 (69,2%)

Comments:
- Only in the presence of anemia.

10) In the first round, most participants (8 out of 13, 62%) agreed that iron supplementation-or increased intake of iron-rich foods-is advisable in adolescents with heavy menstrual bleeding, even in the absence of documented anemia.

Participants noted that the decision should be based on complete blood count testing, but preventing subclinical iron deficiency may improve energy, concentration, and overall wellbeing in this age group. Considering that prolonged menstrual blood loss can reduce iron stores even without anemia, and that supplementation is safe with well-tolerated formulations:

Do you consider it advisable to recommend iron supplementation (or targeted dietary increases) for adolescents with heavy menstrual bleeding, even in the absence of anemia, possibly based on periodic blood count screening?

n*	Slightly not to not at all important (%)	Neutral (%)	Slightly to extremely important (%)
13	0 (0%)	2 (15,4%)	11 (84,6%)

11) In the first round, most participants (8 out of 13, 62%) agreed on the importance of early nutritional intervention (before age 30) to promote women's bone health, while 5 participants remained more cautious.

Comments highlighted the role of calcium-rich mineral waters, aged cheeses, cruciferous vegetables, and the usefulness of avoiding chronic hydration with low-mineral waters. Peak bone mass is achieved by the third decade of life, and early nutritional and behavioural interventions

(e.g., physical activity, adequate intake of calcium, vitamin D and protein) may reduce the risk of osteopenia and osteoporosis later in life.

In light of these considerations, do you consider it appropriate to recommend early nutritional and behavioural interventions (before age 30) to promote bone health and prevent loss of bone mineral density across the lifespan?

n*	Slightly not to not at all important (%)	Neutral (%)	Slightly to extremely important (%)
13	3 (23,1%)	1 (7,7%)	9 (69,2%)

12) In the first round, most participants (8 out of 13, 62%) agreed on the usefulness of personalized nutrition in the management of endometriosis, while 5 participants took a more cautious stance.

Comments stressed the importance of assessing associated gastrointestinal conditions or sensitivities (lactose intolerance, gluten sensitivity, yeast fermentation) and reducing intake of foods with potential inflammatory or pro-estrogenic effects. Personalized nutrition may help control chronic pelvic pain, improve quality of life, and reduce systemic inflammation.

In light of these considerations, do you consider it useful to recommend a personalized nutritional approach in women with endometriosis, aimed at reducing inflammation and improving gastrointestinal symptoms and pain, potentially involving a specialized nutritionist?

n*	Slightly not to not at all important (%)	Neutral (%)	Slightly to extremely important (%)
13	2 (15,4%)	0 (0%)	11 (84,6%)

Comments:

- I consider it useful, but more data are needed.

13) In the first round, most participants (9 out of 13, 69%) agreed on the usefulness of a personalized nutritional approach for managing severe premenstrual syndrome, while a minority remained more cautious.

Comments indicated that dietary modulation may reduce intestinal inflammation, inflammatory cytokine production, and hormonal variability, with potential benefits on pain, irritability, and fluid retention. A targeted dietary approach (reducing refined sugars and pro-inflammatory foods; increasing magnesium, omega-3, and fiber) appears useful and well tolerated.

In light of these considerations, do you consider it useful to recommend personalized nutrition for women with severe premenstrual syndrome, aimed at modulating symptoms through inflammation control and improving metabolic and hormonal balance?

n*	Slightly not to not at all important (%)	Neutral (%)	Slightly to extremely important (%)

13	3 (23,1%)	1 (7,7%)	9 (69,2%)

Comments:

- Same as for endometriosis.

14) In the first round, most participants (8 out of 13, 62%) agreed on the importance of a diet rich in omega-3, antioxidants, and micronutrients for preventing cognitive decline in menopausal women, while others remained neutral.

Recent evidence suggests that such dietary patterns may promote neuroprotection, reduce systemic and oxidative inflammation, improve endothelial function, and support mood and memory during the hormonal transition.

In light of these considerations, do you consider it useful to recommend a diet rich in omega-3 (e.g., oily fish, flaxseed, walnuts), antioxidants (colourful fruits and vegetables, polyphenols), and micronutrients (B-vitamins, magnesium, selenium, zinc) as a preventive measure for cognitive and neurological health in menopausal women?

**n***	**Slightly not to not at all important (%)**	**Neutral (%)**	**Slightly to extremely important (%)**
13	2 (15,4%)	3 (23,1%)	8 (61,5%)

15) In the first round, opinions on DHA supplementation in the postpartum period and during breastfeeding were divided:
6 out of 13 (46%) were in favour (Likert 7-9),
7 out of 13 (54%) were uncertain or opposed (Likert 0-6).

Many comments noted that DHA can be adequately provided through diet (oily fish, seeds, nuts), but that supplementation may be useful in women with low intake of omega-3 sources or in vegetarian/vegan diets, for maternal wellbeing and the infant's neurological development.

In light of these considerations, do you consider it advisable to recommend DHA supplementation in the postpartum period and during breastfeeding (e.g., 200-300 mg/day) in women with low fish consumption or reduced dietary omega-3 intake, as support for maternal health and neonatal cognitive development?

**n***	**Slightly not to not at all important (%)**	**Neutral (%)**	**Slightly to extremely important (%)**
13	4 (30,8%)	0 (0%)	9 (69,2%)

Comments:

- Several studies have shown that omega-3 levels in breast milk improve neurobehavioral outcomes.

16) In the first round, most participants (8 out of 13, 62%) agreed on the usefulness of magnesium supplementation in perimenopausal and menopausal women, while some expressed uncertainty.

Magnesium is involved in numerous biological processes-neuromuscular function, energy metabolism, sleep and mood regulation-and deficiency is relatively common in this life phase due to unbalanced diets, stress, and hormonal changes. Supplementation is generally well tolerated, with potential benefits on fatigue, irritability, insomnia, muscle cramps, and vasomotor symptoms.

In light of these considerations, do you consider it advisable to recommend magnesium supplementation in perimenopausal and menopausal women, even in the absence of documented deficiency, as support for neuromuscular wellbeing, sleep, and mood stability?

n*	Slightly not to not at all important (%)	Neutral (%)	Slightly to extremely important (%)
13	5 (38,5%)	0 (0%)	8 (61,5%)

Comments:

- I do not believe there is supporting evidence.

17) In the first round, opinions on folate-fortified estrogen-progestin contraceptives were evenly split:
6 out of 13 participants (46%) were in favor,
6 out of 13 (46%) were opposed or uncertain.

Some participants reported past negative experiences, while others emphasized that this strategy would be more effective if accompanied by nutritional education and proper preconception counseling. It is known that estrogen-progestin contraceptives may reduce plasma folate levels, and supplementation could help prevent deficiencies in women who discontinue contraception when planning a pregnancy.

In light of these considerations, do you consider the dissemination of folate-fortified estrogen-progestin contraceptives to be advisable as a complementary preventive measure, provided it is accompanied by adequate nutritional education and preconception folate counseling?

n*	Slightly not to not at all important (%)	Neutral (%)	Slightly to extremely important (%)
13	4 (30,8%)	1 (7,7%)	8 (61,5%)

Comments:

- It would not solve the issue, since only a relatively small proportion of women use hormonal contraceptives, and if pregnancy does not occur immediately, the benefit of supplementation may be lost.

18) Iron deficiency is one of the most common nutritional conditions among women of reproductive age, often present even without clinical anemia. Adequate iron status before conception is associated with improved pregnancy outcomes, lower incidence of gestational anemia, and reduced risk of preterm birth and low birth weight.

Since rebuilding iron stores may take several weeks, preconception prevention represents a simple and safe public health measure.

In light of these considerations, do you consider it important to recommend iron supplementation in women who are planning a pregnancy, even in the absence of anemia, to optimize iron stores and reduce the risk of deficiency during gestation?

n*	Slightly not to not at all important (%)	Neutral (%)	Slightly to extremely important (%)
13	5 (38,5%)	1 (7,7%)	7 (53,8%)

Comments:

- Iron, in the absence of iron deficiency, may have pro-inflammatory effects.

19) In the first round, there was strong agreement on the importance of folic acid (vitamin B9) supplementation in the preconception period. However, recent evidence suggests that vitamins B6 and B12 also play a crucial role in regulating plasma homocysteine levels, whose elevation is associated with increased risk of vascular complications, early pregnancy loss, and neural tube defects.
A deficiency in any one of these vitamins can impair the metabolic effectiveness of the others; therefore, combined supplementation (B6, B9, B12) is more effective in maintaining adequate methylation balance and supporting reproductive health.

In light of these considerations, do you consider it important to recommend combined B-vitamin supplementation (B6, B9, B12) in the preconception period, rather than folic acid alone, in order to optimize homocysteine levels and reduce the risk of reproductive complications?

n*	Slightly not to not at all important (%)	Neutral (%)	Slightly to extremely important (%)
13	4 (30,8%)	2 (15,4 %)	7 (53,8%)

Comments:

- I am not aware of evidence supporting this.
- It is important to clarify that folic acid is not vitamin B9, but a chemical precursor, and that in individuals with elevated (>12) or suboptimal (>7) homocysteine levels, MTHFR hetero- or homozygosity is likely, implying reduced ability to convert folic acid into active vitamin B9.

20) First-round comments highlighted the role of micronutrients in glucose and hormonal metabolism, particularly in conditions such as PCOS, gestational diabetes, or insulin resistance. Recent evidence indicates that chromium-especially in picolinate or nicotinate forms-may improve insulin sensitivity and glycaemic control, reducing insulin production and systemic inflammation. Its use at low doses (‚â§200 ¬µg/day) is considered safe and potentially beneficial as complementary nutritional support.

In light of these considerations, do you consider chromium supplementation useful as nutritional support for improving glucose metabolism in women with PCOS?

n*	Slightly not to not at all important (%)	Neutral (%)	Slightly to extremely important (%)
13	7 (53,8%)	2 (15,4%)	4 (30,8%)

Comments:

- I am not familiar with this topic.

21) Do you consider chromium supplementation useful as nutritional support for improving glucose metabolism in women with gestational diabetes?

n*	Slightly not to not at all important (%)	Neutral (%)	Slightly to extremely important (%)
13	7 (53,8%)	3 (23,1%)	3 (23,1%)

Comments:

- I am not familiar with these data.

22) Do you consider chromium supplementation useful as nutritional support for improving glucose metabolism in women with insulin resistance?

n*	Slightly not to not at all important (%)	Neutral (%)	Slightly to extremely important (%)
13	7 (53,8%)	1 (7,7%)	4 (38,5%)

Comments:

- I am not familiar with these data.
- Chromium is naturally present in many food groups. In women with insulin resistance, I have not found strong evidence supporting supplementation. It may perhaps be useful in women with type 2 diabetes who are unable to follow nutritional and physical activity recommendations.
